# Obesity, Diabetes, and Cardiovascular Risk Burden in Systemic Lupus Erythematosus: Current Approaches and Knowledge Gaps—A Rapid Scoping Review

**DOI:** 10.3390/ijerph192214768

**Published:** 2022-11-10

**Authors:** Halbert Hernández-Negrín, Michele Ricci, Juan José Mancebo-Sevilla, Jaime Sanz-Cánovas, Almudena López-Sampalo, Lidia Cobos-Palacios, Carlos Romero-Gómez, Iván Pérez de Pedro, María del Mar Ayala-Gutiérrez, Ricardo Gómez-Huelgas, María Rosa Bernal-López

**Affiliations:** 1 Internal Medicine Clinical Management Unit, Hospital Regional Universitario de Málaga, Instituto de Investigación Biomédica de Málaga (IBIMA-Plataforma BIONAND), Avenida Carlos Haya S/N, 29010 Malaga, Spain; 2 Faculty of Medicine, Universidad de Málaga, Campus Teatinos, 29010 Malaga, Spain; 3 Centro de Investigación Biomédica en Red Fisiopatología de la Obesidad y Nutrición (CIBERobn), Instituto de Salud Carlos III, 28029 Madrid, Spain

**Keywords:** cardiovascular risk, diabetes mellitus, obesity, systemic lupus erythematosus

## Abstract

Obesity, diabetes mellitus, and cardiovascular risk are real challenges in systemic lupus erythematosus (SLE) clinical practice and research. The evidence of the burden of these health problems in SLE patients is determined by the methods used to assess them. Therefore, the aim of this scoping review is to map current approaches in assessing obesity, diabetes mellitus, and cardiovascular risk burden in SLE patients and to identify existing knowledge gaps in this field. This rapid scoping review was conducted according to the Joanna Briggs Institute methodology and identified 274 articles, of which 73 were included. Most studies were conducted at European institutions and patients were recruited from specialist hospital clinics, the majority of whom were women. The burden of obesity and diabetes mellitus for SLE patients was assessed mainly in terms of prevalence, impact on disease activity, and cardiometabolic risk. The burden of cardiovascular risk was assessed using multiple approaches, mainly imaging and laboratory methods, and risk factor-based scores, although there is great heterogeneity and uncertainty between the methods used. This review highlights the importance of improving and standardizing the approach to obesity, diabetes, and cardiovascular risk in SLE patients through a holistic assessment that includes lifestyle, clinical, biological, and social aspects.

## 1. Introduction

Despite improvements in health outcomes in systemic lupus erythematosus (SLE) in recent years, these patients experience higher mortality than the general population [1]. One of the main causes of death in these patients is cardiovascular disease. The excess of cardiovascular disease in SLE patients is not fully explained by a higher prevalence of classical cardiovascular risk factors [2,3]. Numerous efforts have been made to understand the pathogenesis behind this phenomenon and to develop therapeutic strategies that allow adequate management of cardiovascular disease in this group [3,4]. However, at present, only the control of classic cardiovascular risk factors and disease activity with hydroxychloroquine and the lowest possible glucocorticoid dosage are the standard recommendations [5,6,7].

In this context, an adequate assessment of obesity, diabetes, and cardiovascular risk burden is particularly relevant for these patients and society. Systematic reviews have addressed this question by providing evidence of obesity and cardiovascular risk burden in SLE patients. The prevalence of obesity in SLE patients is very high, ranging from 28% to 50% depending on the measurement methods [8]. It appears that obesity is associated with more severe cognitive and renal involvement, alteration of the quality of life, and contributes to the enhanced cardiovascular risk in SLE patients [8]. Additionally, SLE patients exhibit a high cardiovascular risk, having up to 3 times more risk of developing cardiovascular disease than the general population [2,9,10,11]. Systematic reviews focused on the impact of diabetes mellitus in SLE patients were not found in scientific literature.

The evidence of the burden of these health problems in SLE patients is shaped by the methods used to assess them. In this regard, several approaches have emerged in recent years, ranging from molecular to imaging methods, including equations and indexes which do not always perform adequately or are useful in clinical practice [12,13,14]. The lack of consensus and the great heterogeneity in the way assessment methods are applied and interpreted has consolidated obesity, diabetes mellitus, and cardiovascular risk as a real challenge for SLE research and clinical practice [12,15].

Given these facts, it would be useful to know how current research is being conducted and what kind of evidence is available on this topic, as well as what are the gaps in current knowledge. To answer these questions, the best source is a scoping review that synthesizes and maps the evidence, while clarifying key concepts that can serve as a basis for future research development [16].

However, after searching MEDLINE, the Cochrane Database of Systematic Reviews, and JBI Evidence Synthesis, no current or ongoing scoping reviews on the topic were identified. Therefore, the aim of this scoping review is to map current approaches in assessing obesity, diabetes mellitus, and cardiovascular risk burden in SLE patients and to identify existing knowledge gaps in this field.

## 2. Materials and Methods

### 2.1. Protocol and Registration

To develop this rapid scoping review, the Joanna Briggs Institute (JBI)methodology [16] was followed and the Preferred Reporting Items for Systematic Reviews and Meta-Analysis extension for scoping reviews guidelines (PRISMA-ScR) were fulfilled [17]. A protocol was developed but not registered.

The following two research questions were posed: 1. What is known from the literature about current approaches to assess obesity, diabetes, and cardiovascular risk burden in SLE? and 2. What are the current knowledge gaps in this field?

### 2.2. Eligibility Criteria

Inclusion criteria were defined according to JBI recommendations for scoping reviews, based on “Population”, “Concept” and “Context”:

Population: Studies that include SLE human patients older than 18 years were considered for this review.

Concept: Studies that evaluate the obesity, diabetes mellitus, and cardiovascular risk burden were included. In the case of obesity and diabetes mellitus, only those studies that assessed at least one measure of burden other than the prevalence of obesity and/or diabetes in SLE patients were included. The burden was interpreted as the association of obesity and/or diabetes mellitus with SLE-specific and non-SLE-specific health outcomes. In the case of cardiovascular risk, we included those studies in which the authors stated the cardiovascular risk assessment method in SLE patients, regardless of the method used.

Context: For this review, studies were considered regardless of the country where they were conducted or the setting.

Types of studies: This review included peer-reviewed studies regardless of whether their design was quantitative, qualitative, or mixed, with the aim of capturing different approaches to disease burden assessment. Reviews, systematic reviews, conference abstracts, case reports, opinion articles, and gray literature were not considered.

### 2.3. Information Sources

Relevant papers were screened by searching the following bibliographic databases: MEDLINE and EMBASE. Since this was a rapid scoping review, it was not possible to extract relevant bibliography from the reference lists of the included articles.

### 2.4. Search Strategy

The search strategy was aimed at identifying original articles published between 2017 and April 2022. The search strategy was developed by an experienced librarian and refined through team discussion. The final search strategy for MEDLINE is available in Table 1, this strategy was adapted to EMBASE database.

To ensure an accurate evaluation of the literature and quality data extraction, only those studies published in the languages in which the authors are fluent (English and Spanish) were included.

### 2.5. Selection of Sources of Evidence

Once the search was completed, all identified citations were collated and loaded into EndNote 7.8/2016 (Clarivate Analytics, Philadelphia, PA, USA) and duplicates were removed. After a pilot test, titles and abstracts were examined by two independent reviewers to assess them against the review inclusion criteria. The full text of selected citations was evaluated in detail against the inclusion criteria by two independent reviewers. In cases where the full text was not available, the authors were contacted. Disagreements arising between reviewers at each stage of the selection process were resolved through discussion, or with a third reviewer. The results of the search and study inclusion process are reported in a flow chart of the PRISMA-ScR.

### 2.6. Data Charting Process

Data were extracted from the included articles by two independent reviewers using a data extraction tool developed by the authors. Extracted data included pertinent details about the participants, the concept, the context, the study methods, and the key findings relevant to the review question.

### 2.7. Synthesis of Results

The general characteristics of the included studies were synthesized using absolute frequencies and are presented in a table. The approaches used to assess the burden were analyzed independently in three groups: obesity, diabetes mellitus, and cardiovascular risk. The main methods identified were categorized and presented in a figure. The knowledge gaps found in the review were developed in narrative form in the text. Critical appraisal was not performed as it was not the objective of this research.

## 3. Results

### 3.1. Selection of Sources of Evidence

**Figure 1:**
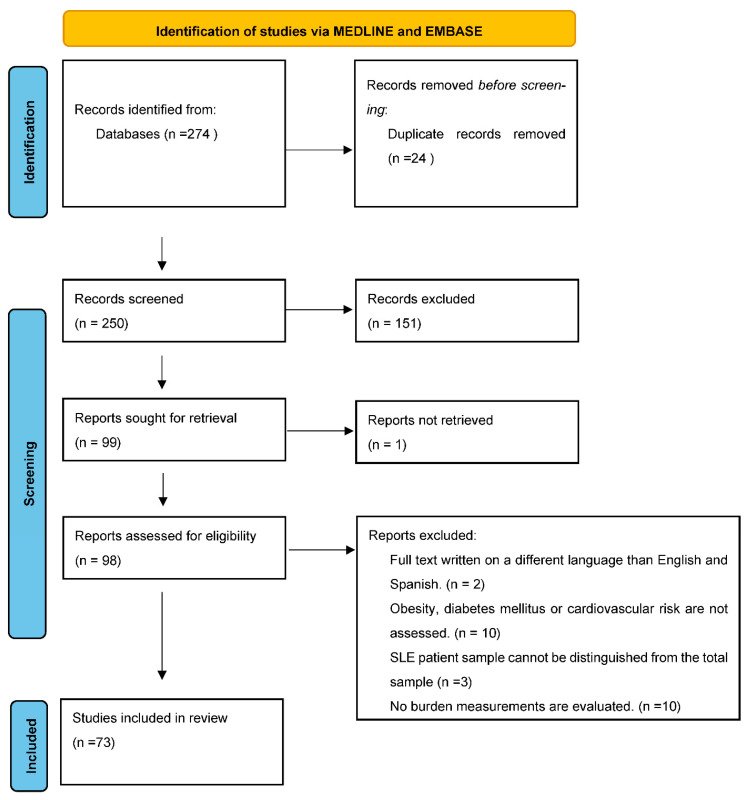
PRISMA flow chart of the article selection process.

The search resulted in 274 references. After excluding duplicates and weighing titles, abstracts, and full text according to the inclusion criteria, we finally included 73 articles in this review. Figure 1 shows the screening process in a PRISMA flowchart.

### 3.2. Characteristics of Sources of Evidence

The general characteristics of the included studies related to year of publication, geographic region, type of study reported by the authors, number of participants, percentage of women, and time frame are summarized in Table 2.

Most of the included studies were published in 2019 and represented SLE patients from Europe, although patients from 24 countries were included. The country that contributed to the most studies was Spain (n = 17), followed by the USA (n = 9).

Most of the studies were developed with samples of SLE patients recruited from specialized hospital clinics. More than half of the studies were able to recruit more than 100 patients with SLE. In 73% of the studies, the sample was composed of more than 90% of SLE female patients. In 50% of the studies, the authors reported a cross-sectional design and the time frame of the investigations in most cases was between 1 and 5 years.

### 3.3. Results of Individual Sources of Evidence

For each source of evidence included, the relevant results to the objective of this review are shown in the Supplementary Materials. All studies included in the obesity and diabetes mellitus group evaluated their prevalence (%) in SLE patients.

### 3.4. Synthesis of Results

**Figure 2:**
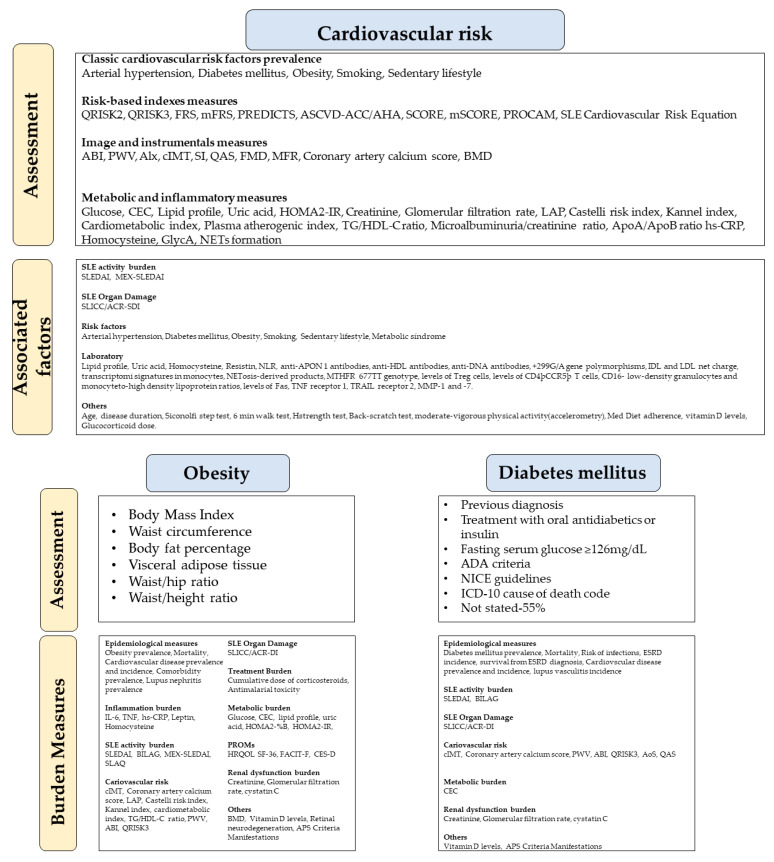
Current approaches to assessing obesity, diabetes mellitus, and cardiovascular risk burden in SLE patients. (ABI: Ankle Brachial Index. ADA: American Diabetes Association. Alx: Augmentation index. AoS: Aortic stiffness. APS: Antiphospholipid syndrome. ASCVD-ACC/AHA: Pooled Cohort Risk Equations-American College of Cardiology/American Heart Association. BILAG: British Isles Lupus Assessment Group. BMD: Bone mineral density. CEC: Cholesterol Efflux Capacity. CES-D: Center for Epidemiological Studies-Depression. cIMT: Carotid intima-media thickness. CVD: Cardiovascular disease. ESRD: End-stage renal disease. FACIT-F: Functional Assessment of Chronic Illness Therapy—Fatigue. FMD: Flow-mediated dilation. FRS: Framingham Risk Score. GlycA: Glycoprotein acetylation. HOMA-IR: Homeostatic Model Assessment of Insulin Resistance. HRQOL-SF 36: Health-related quality of life -The Short Form-36 Health Survey. hs-CRP: High-sensitive C-reactive protein. ICD-10: International Classification of Diseases 10th Revision. IDL: Intermediate density lipoproteins. IL-6: Interleukin-6. LAP: Lipid accumulation product. LDL: low-density lipoprotein. Med Diet: Mediterranean diet. MEX-SLEDAI: Mexican Systemic Lupus Erythematosus Disease Activity Index. MFR: Myocardial flow reserve. mFRS: Modified Framingham Risk Score. MMP-1: Collagenase-1. MMP-7: Matrilysin. mSCORE: Modified Systematic Coronary Risk Evaluation. NETs: Neutrophil extracellular traps. NICE: National Institute for Health and Care Excellence. NLR: Neutrophil-to-lymphocyte ratio. PGA: Physician Global Assessment score. PON1: Serum paraoxonase and arylesterase 1. PREDICTS: Predictors of Risk for Elevated Flares, Damage Progression and Increased Cardiovascular Disease in Patients with SLE. PROCAM: Prospective Cardiovascular Münster Study risk calculator. PWV: Pulse wave velocity. QAS: quality arterial stiffness. QRISK3: QRESEARCH risk estimator, version 3. SCORE: Systematic Coronary Risk Evaluation. SLAQ: Systemic Lupus Activity Questionnaire. SLEDAI: Systemic Lupus Erythematosus Disease Activity Index. SLICC/ACR-DI: Systemic Lupus International Collaborating Clinics/American College of Rheumatology-Damage index. TG/HDL-C: Serum levels of triglyceride/high-density lipoprotein cholesterol ratio. TNF: tumor necrosis factor. TRAIL: TNF-related apoptosis-inducing ligand).

The main methods identified to assess the burden of obesity, diabetes mellitus, and cardiovascular risk in SLE patients are categorized and presented in Figure 2.

An important knowledge gap lies in the fact that it is not possible to ensure a causal relationship between the measures studied and the results, due to the methodological limitations of the study designs. Moreover, the influence of individual and contextual socioeconomic factors that may be shaping the impact of obesity, diabetes, and cardiovascular risk in SLE patients is largely unknown. Due to the marked predominance of female patients and the large number of European studies conducted, the burden of previously mentioned factors and the methods to assess them in male SLE patients as well as those living in other continents (South America, Africa, and Asia) has been poorly studied. On the other hand, the impact of obesity, diabetes mellitus, and cardiovascular risk on treatment outcomes, disability, health care utilization, and costs have been scarcely studied.

In summary, the magnitude and nature of the relationship between obesity, diabetes, and cardiovascular risk in SLE remain poorly understood. Although new methods have been developed and others refined, a specific strategy for cardiovascular risk stratification in these patients remains to be defined. Even if this milestone is achieved, the challenge remains to extrapolate the results to SLE patient populations that continue to be underrepresented in research in this area. Finally, there are few studies based on implementation science to evaluate these methods in daily clinical practice and to test the feasibility and effectiveness of different approaches.

## 4. Discussion

In this rapid exploratory review, we included 73 studies published between 2017 and April 2022 that evaluated the obesity, diabetes mellitus, and cardiovascular risk burden in patients with SLE.

Our results indicate that there is little recent evidence regarding the impact of diabetes mellitus on SLE patients. This could be influenced by the fact that many studies of cardiovascular risk exclude patients who already have diabetes mellitus. We also found multiple studies that evaluated the burden of obesity and cardiovascular risk in this group, although many studies evaluated both at the same time.

In the case of obesity, several methods were used to assess it, of which BMI was the most widespread and accessible. However, probably the measurement of body fat index by DXA is the most robust method that overcomes the limitations of BMI [19]. Despite this, there is little evidence comparing the performance of both methods in patients with SLE [20].

The greatest heterogeneity in the approaches used to evaluate the burden was found in the assessment of cardiovascular risk, where the authors used classical cardiovascular risk factors to modern imaging and molecular techniques, although many need validation in longitudinal studies [6,21,22,23,24,25]. We consider that this heterogeneity is due to the varied conditions in the different scenarios in which SLE clinical practice and research are developed. In the absence of a robust recommendation applicable to all patients, the assessment of individual cardiovascular risk relies on the judgment of physicians and their experience in the application of risk scores and laboratory and imaging methods [26].

The incorporation of SLE and the use of corticosteroids in risk scores seems to be a step in the right direction to establish a more accurate cardiovascular risk stratification tool for patients with SLE, although further optimization, standardization, and simplification are needed for use in daily practice [6,13,14,26,27].

In general, when evaluating the impact of the health problems studied in SLE patients, there was a lack of consideration and adjustment for socioeconomic factors [28]. Socioeconomic data are often easy to obtain, but researchers should be aware of their importance as they may modify the impact of obesity, diabetes mellitus, and cardiovascular risk in underserved SLE patient populations. An interesting alternative would be to develop a cardiovascular risk model for SLE patients that takes into account the increased risk associated with social deprivation, as does the QRISK scale.

Our results revealed that there are certain measures of burden that have been poorly evaluated in recent literature, such as the burden of treatment, disability, health care utilization, work participation, and, clearly, economic burden [13,28]. Most studies to date that have attempted to assess the impact of obesity and diabetes on the health outcomes of SLE patients have a cross-sectional design, so causality cannot be inferred [15,19,29,30,31]. This has determined that the role of obesity and diabetes in SLE remains poorly understood [19].

More than 50% of the included studies had a sample composed of more than 90% women. This finding was not surprising because of the differences in the prevalence of SLE according to sex. However, atherosclerosis affects men and women differently regarding incidence, prevalence, risk factors, pathogenesis, clinical manifestations, treatment, morbidity, and mortality. For optimal prevention and treatment of atherosclerosis, it is not self-evident that women and men show similar responses to risk factors or treatment; therefore, studies must present results according to sex. Whether the threat and risk factors of premature atherosclerosis in women with SLE are generalized to male patients is unknown [3,26,31].

In addition to the underrepresentation of male patients, the included studies had little ethnic diversity in their sample [3,13,32]. This fact, combined with only a minority of the studies being conducted in South America, Africa, and Asia, creates uncertainty about how obesity, diabetes mellitus, and cardiovascular risk are being assessed in these SLE patient populations and limits the extrapolation of results obtained from research conducted in other settings.

Considering the personalized care of patients, the potential impact of the clinical phenotype, cardiometabolic profile, and social context of each SLE patient on cardiovascular prognosis deserves further investigation [6].

Our rapid exploratory review has some limitations. We only performed a systematic search in two bibliographic databases, so we recognize that it is possible that studies of relevance to our research question may have been left out. Although the initial protocol included a search of the references of the selected articles in search of new studies that met our inclusion criteria, this process could not be carried out due to time constraints. An evidence quality appraisal was not performed as this was not in line with the objectives of this rapid exploratory review. However, to the best of our knowledge, this is the first scoping review that provides a panoramic and updated view on this topic and points out important gaps in knowledge in the area that serves as a basis for the development of future research.

## 5. Conclusions

Despite the evident interest of clinicians and researchers in obesity, diabetes mellitus, and cardiovascular risk in SLE, there is great heterogeneity and uncertainty in the methods used to assess their burden. Future research with robust methodological designs is needed to compare the different novel methods with those currently available in terms of their validity and usefulness in clinical practice.

Our rapid scoping review has captured the disparity in scientific production in this regard, where male SLE patients, as well as Asians, Africans, and South Americans are underrepresented. This fact limits the extrapolation of the existing scientific evidence to these groups and indicates lines of future research in this area.

Furthermore, our review highlights the importance of improving and standardizing the approach to obesity, diabetes, and cardiovascular risk in patients with SLE through a holistic assessment that includes lifestyle, clinical, biological, and social aspects. We believe that only in this way will we be able to understand, assess, and intervene in the complex mechanisms underlying the relationship between SLE, obesity, diabetes, and cardiovascular risk.

## Figures and Tables

**Table 1:** Search strategy for MEDLINE (PubMed). The search was conducted on 3 May 2022.

Search	Query	Record Retrieved
#1	((“lupus erythematosus, systemic”[MeSH Terms] OR “lupus erythematosus systemic”[Title/Abstract]) AND (“Obesity”[MeSH Terms] OR “Obesity”[Title/Abstract])) AND ((humans[Filter]) AND (2017/1/1:2022/4/30[pdat]) AND (english[Filter] OR spanish[Filter]) AND (alladult[Filter]))	43
#2	((“lupus erythematosus, systemic”[MeSH Terms] OR “lupus erythematosus systemic”[Title/Abstract]) AND (“Diabetes Mellitus”[MeSH Terms] OR “Diabetes Mellitus”[Title/Abstract])) AND ((humans[Filter]) AND (2017/1/1:2022/4/30[pdat]) AND (english[Filter] OR spanish[Filter]) AND (alladult[Filter]))	73
#3	((“lupus erythematosus, systemic”[MeSH Terms] OR “lupus erythematosus systemic”[Title/Abstract]) AND “Cardiovascular Risk”[Title/Abstract]) AND ((humans[Filter]) AND (2017/1/1:2022/4/30[pdat]) AND (english[Filter] OR spanish[Filter]) AND (alladult[Filter]))	103

**Table 2:** Study characteristics.

Study Characteristics	No of Studies	Study Characteristics	No of Studies
Year of Publication		Number of Participants	
2017	9	<50	10
2018	13	50–100	22
2019	14	101–150	16
2020	16	>150	25
2021	19		
2022 *	2		
Geographic region		Type of study reported by the authors	
Europa	37	Cross-sectional	37
North America	11	Cohort	10
South America	9	Prospective cohort	7
Africa	7	Retrospective cohort	3
Asia	7	Case-control	2
Oceania	1	Clinical trial	2
Multi-site **	1	Retrospective study	2
Setting		Prospective study	1
Specialized hospital clinic	67	Not stated	9
Hospitalized patients	1		
Population-based registries	3		
Both clinical- and community-based sources	2	Time frame	
Percentage of women		<1 year	8
<90	18	1–5 years	28
90–100	54	>5 years	13
Not stated	1	Not stated	24

* In 2022, studies published up to April were included. ** One of the included studies was an international multicenter inception cohort involving 33 centers across North America, Europe, and Asia [18].

## Data Availability

The data presented are available upon request from the corresponding author.

## Supplementary Materials

The following supporting information can be downloaded at: https://www.mdpi.com/article/10.3390/ijerph192214768/s1, Table S1: Characteristics of individual evidence sources related to obesity. Table S2: Characteristics of individual evidence sources related to diabetes mellitus. Table S3: Characteristics of individual evidence sources related to cardiovascular risk. References [33,34,35,36,37,38,39,40,41,42,43,44,45,46,47,48,49,50,51,52,53,54,55,56,57,58,59,60,61,62,63,64,65,66,67,68,69,70,71,72,73,74,75,76,77,78,79,80,81,82,83,84,85] are cited in the Supplementary Materials.
