# Supplementary material for: Obesity, Diabetes, and Cardiovascular Risk Burden in Systemic Lupus Erythematosus: Current Approaches and Knowledge Gaps—A Rapid Scoping Review
Source: Int J Environ Res Public Health. 2022 Nov 10;19(22):14768. doi: 10.3390/ijerph192214768 (PMC9690813; doi:10.3390/ijerph192214768)
Supplement: Supplementary file 1 [file ijerph-19-14768-s001.zip › ijerph-1920526-supplementary.pdf]

Abbreviations

ABI: Ankle Brachial Index
ADA: American Diabetes Association
Alx: Augmentation index
AoS: Aortic stiffness
APS: Antiphospholipid syndrome
ASCVD-ACC/AHA: Pooled Cohort Risk Equations-American College of Cardiology/American Heart Association
BILAG: British Isles Lupus Assessment Group
BMD: Bone mineral density
CEC: Cholesterol Efflux Capacity
CES-D: Center for Epidemiological Studies-Depression
cIMT: Carotid intima-media thickness
CVD: Cardiovascular disease
ESRD: End-stage renal disease
FACIT-F: Functional Assessment of Chronic Illness Therapy – Fatigue
FMD: Flow-mediated dilation
FRS: Framingham Risk Score
GlycA: Glycoprotein acetylation
HOMA-IR: Homeostatic Model Assessment of Insulin Resistance
HRQOL-SF 36: Health-related quality of life -The Short Form-36 Health Survey
hs-CRP: High sensitive C-reactive protein
ICD-10: International Classification of Diseases 10th Revision.
IDL: Intermediate density lipoproteins
IL-6: Interleukin-6
LAP: Lipid accumulation product
LDL: low-density lipoprotein
Med Diet: Mediterranean diet
MEX-SLEDAI: Mexican Systemic Lupus Erythematosus Disease Activity Index
MFR: Myocardial flow reserve
mFRS: Modified Framingham Risk Score
MMP-1: Collagenase-1
MMP-7: Matrilysin
mSCORE: Modified Systematic Coronary Risk Evaluation
NETs: Neutrophil extracellular traps
NICE: National Institute for Health and Care Excellence
NLR: Neutrophil-to-lymphocyte ratio
N/S: Not stated
PGA: Physician Global Assessment score
PON1: Serum paraoxonase and arylesterase 1
PREDICTS: Predictors of Risk for Elevated Flares, Damage Progression and Increased Cardiovascular Disease in Patients with SLE
PROCAM: Prospective Cardiovascular Münster Study risk calculator
PWV: Pulse wave velocity
QAS: quality arterial stiffness
QRISK3: QRESEARCH risk estimator, version 3
SCORE: Systematic Coronary Risk Evaluation
SLAQ: Systemic Lupus Activity Questionnaire
SLEDAI: Systemic Lupus Erythematosus Disease Activity Index
SLICC/ACR-DI: Systemic Lupus International Collaborating Clinics/American College of Rheumatology-Damage index
TG/HDL-C: Serum levels of triglyceride/ high-density lipoprotein cholesterol ratio.
TNF: tumor necrosis factor
TRAIL: TNF-related apoptosis-inducing ligand

Table S1: Characteristics of individual evidence sources related to obesity.

Source	Year of publication	Country	Setting	Type of study reported by the authors	Time frame	Number of participants	Percentage of women	Obesity assessment	Obesity definition	Burden measures
Pesqueda-Cendejas K et al[39]	2022	Mexico	Specialized hospital clinic	Cross-sectional	3 years	176	100	BMI<br>Waist-hip ratio<br>Waist-height ratio<br>Fat mass	World Health Organization criteria	hs-CRP
Sola Rodríguez S et al[30]	2021	Spain	Specialized hospital clinic	Cross-sectional	5 months	77	100	BMI<br>Waist circunference<br>Body fat percentage<br>Waist-height ratio<br>Waist-hip ratio	N/A	hs-CRP and<br>IL-6<br>Leptin
Gergianaki I et al [69]	2021	Greece	Population-based registries	Cohort	44 month	399	91.2	BMI	World Health Organization criteria	BILAG
Shaaban A et al[37]	2021	Egypt	Specialized hospital clinic	Cross sectional	N/S	30	76.7	BMI<br>Waist circumference<br>waist-to-hip ratio	N/S	cIMT
Tsyhanyk L et al[76]	2021	Ukraine	Specialized hospital clinic	N/S	N/S	123	100	BMI	N/S	BMD
Sola-Rodriguez S et al[31]	2021	Spain	Specialized hospital clinic (multicentric)	Cross-sectional	5 month	77	100	BMI	N/S	Clustered cardiometabolic risk: Systolic blood pressure, fasting glucose,<br>TG, HOMA-IR, TC/HDL-c, and hs-CRP

Borg A et al[28]	2021	Sweden	Specialized hospital clinic (Multicentric)	Clinical trials	13 months	1684	94.1	BMI	World Health Organization criteria	HRQoL-SF-36<br>FACIT-F
Pedrosa T et al[77]	2021	Brazil	Specialized hospital clinic	Cross-sectional	N/S	108	91.6	BMI	World Health Organization criteria	Risk of HCQ toxicity
Chanloung W et al[67]	2021	Thailand	Specialized hospital clinic	Cross-sectional	2 years	132	87	BMI	BMI >25 kg/m2	APS Criteria Manifestations
Campos-Lopez B et al[55]	2021	Mexico	Specialized hospital clinic	Cross-sectional	2 years	158	100	BMI<br>Waist circunference<br>Waist-height ratio<br>Waist-hip ratio	World Health Organization criteria	SLICC-ACR DI<br>Mex-SLEDAI<br>hs-CRP<br>Glucose<br>Castelli index<br>Kannel index<br>TG/HDL-C ratio<br>High TG/ low HDL-C<br>Cardiometabolic index<br>LAP score
Gomez A et al[79]	2021	Sweden	Specialized hospital clinic (Multicentric)	Cross-sectional	13 months	1684	94.1	BMI	World Health Organization criteria	HRQoL-SF-36<br>EQ-5D<br>FACIT-F<br>Cumulative prednisolone dosage<br>Intravenous cyclophosphamide, among others<br>Comorbidities prevalence
Dias-Santos A et al[74]	2020	Portugal	Specialized hospital clinic	Cross-sectional	1 year	68	85.2	BMI	N/S	Retinal neurodegeneration
Sanchez-Perez H et al [64]	2020	Spain	Specialized hospital clinic (Multicentric)	Cross-sectional	N/S	195	95	BMI<br>Waist circumference	N/S	CEC<br>cIMT
Ajeganova S et al[65]	2020	Sweden	Specialized hospital clinic	Prospective cohort	7 years	77	90	BMI<br>Waist circumference	World Health Organization criteria	cIMT

Apostolopoulos D et al[78]	2020	Australia	Specialized hospital clinic	Cross-sectional	15 years	116	87.9	BMI	World Health Organization criteria	SLICC/ACR-DI<br>SLEDAI-2K<br>Resistin<br>Lipocalin-2<br>TNF<br>MCP-1<br>IL-6
Kang JH et al[80]	2020	Republic of Korea	Specialized hospital clinic (multicentric)	Prospective cohort	3 years	393	N/S	BMI<br>Waist circumference<br>Waist to hip ratio	World Health Organization criteria	Clinical manifestations prevalence<br>SLEDAI-2K<br>PGA<br>SLICC/ACR-DI<br>HRQoL-SF-36<br>Comorbid depression<br>Newly developed lupus nephritis
Przywara-Chowaniec B et al[49]	2020	Poland	Specialized hospital clinic	N/S	45 month	38	89.4	BMI	N/S	SLEDAI
Pocovi-Gerardino G[63]	2020	Spain	Specialized hospital clinic (Multicentric)	Cross-sectional	22 month	139	90.6	BMI	N/S	hs-CRP<br>Homocysteine
Alian S et al[72]	2019	Egypt	Specialized hospital clinic	Retrospective cohort	N/S	152	88.2	BMI	N/S	Mortality
Behiry ME et al[73]	2019	*Egypt*	Specialized hospital clinic	Cross-sectional	2 month	65	100	BMI	World Health Organization criteria	SLEDAI
Rodriguez M[42]	2019	Spain	specialized hospital clinic	Cross-sectional	N/S	78	92.3	BMI	World Health Organization (WHO) criteria	cIMT
Mellor-Pita S et al[46]	2019	Spain	Specialized hospital clinic	Cross-sectional	N/D	47	100	BMI<br>Waist circunference	N/S	Vitamin D levels

Quevedo-Abeledo JC et al[47]	2019	Spain	Specialized hospital clinic (Multicentric)	Cross-sectional	N/S	276	95	BMI<br>Waist circunference	N/S	cIMT
Li Z et al[20]	2019	China	Specialized hospital clinic	Cross-sectional	3 years	98	100	BMI<br>Fat mas<br>Bone mineral content<br>Visceral Adipose Tissue volume<br>Visceral Adipose Tissue mass<br>Fat mass index<br>Fat-free mass	N/S	SLEDAI-2K<br>SLICC/ACR-DI<br>Lipid profile<br>C3/C4 serum levels<br>hsCR<br>Serum antibody level (ANA, anti-dsDNA, Anti-Smith, anti-Ro, Anti-La)<br>Current or cumulative glucocorticoid dose<br>Lipid profile<br>Serum creatinin<br>Uric acid<br>hs-CRP
Fernandez-Garces M et al[50]	2019	Spain	Specialized hospital clinic (Multicentric)	Cross-sectiona	10 years	335	100	BMI<br>Waist circumference	World Health Organization criteria	CVD
Meza-Meza MR[81]	2019	Mexico	Specialized hospital clinic	Cross-sectional	2 years	130	100	BMI	World Health Organization criteria	Mex-SLEDAI<br>Glucose<br>Lipid profile<br>Blood pressure
Sola-Rodriguez S[29]	2019	Spain	Specialized hospital clinic (Multicentric)	Cross-sectional	5 months	77	100	BMI<br>Waist circunference<br>Body fat percentage<br>Waist-height ratio<br>Waist-hip ratio	N/S	Siconolfi step test<br>6 min walk test<br>handgrip strength test<br>30 s chair stand<br>back-scratch test

Teh P et al[15]	2019	USA	Specialized hospital clinic (Multicentric)	Population-based registries	3 years	137	92	BMI	World Health Organization criteria	SLEDAI<br>Current steroid use<br>Clinical manifestations prevalence (%)
Patterson SL et al[19]	2019	USA	Clinical- and community-based sources	Cross-sectional	N/S	138	100	BMI<br>Fat mass index	Fat mass index ≥ 13 kg/m2 and World Health Organization criteria	SLAQ<br>CES-D<br>SF-36 Pain Subscale<br>SF-36 Vitality Subscale
Yang XF et al [43]	2018	China	Specialized hospital clinic	Cross-sectional	10 month	90	100	BMI	N/S	baPWV
Baragetti A et al[21]	2018	Italy	Specialized hospital clinic	Prospective cohort	5 years	40	90	BMI<br>Waist-hip ratio	N/S	cIMT<br>SLEDAI-2K<br>SLICC/ACR DI<br>PGA
Seguro LPC et al[75]	2018	Brazil	Specialized hospital clinic	N/S	N/S	63	100	BMI<br>Visceral adipose tissue	N/S	CRP<br>HDL<br>LDL<br>TC<br>TG<br>Cumulative glucocorticoid dose
Wigren M et al[22]	2018	Sweden	Specialized hospital clinic	Cohort	9 years	470	88	BMI	N/S	cIMT<br>CVD
Vazquez-Del Mercado M[12]	2018	Mexico	Specialized hospital clinic	N/S	18 month	67	17.9	BMI<br>Waist circunference	World Health Organization criteria	QRISK3<br>cIMT<br>PWV<br>cfPWV<br>Artery Distensibility
Garcia-Carrasco M[45]	2018	Mexico	Specialized hospital clinic	Cross-sectional	18 month	66	100	BMI<br>Waist circunference	N/S	cIMT
Hammam N et al[82]	2018	Egypt	Specialized hospital clinic	Cross-sectional	2 years	74	87.8	BMI	N/S	SLEDAI-2K

								Waist circumference		
Romero-Diaz J et al[3]	2018	Mexico	Specialized hospital clinic	N/S	7 years	95	0	BMI<br>Waist circumference	N/S	Coronary artery calcium score
Tziomalos K et al[33]	2017	Greece	Specialized hospital clinic	N/S	2 years	55	87.3	BMI<br>Waist circunference<br>Waist/hip ratio	N/S	PWV<br>ABI
Sanchez-Perez H et al[38]	2017	Spain	Specialized hospital clinic	Cross-sectional	5 years	87	95	BMI<br>Waist circunference	N/S	HOMA2B%-C-peptide<br>HOMA2-IR
Garcia-Garcia P et al[35]	2017	Spain	Specialized hospital clinic	Cross-sectional	N/S	61	100	BMI	N/S	Cystatin C
Mak A et al[83]	2017	Singapore	Specialized hospital clinic	Case-control	N/S	71	91.6	BMI	N/S	FMD<br>cIMT
Tektonidou MG et al [84]	2017	Greece	Specialized hospital clinic	N/S	2 years	115	92	BMI	N/S	cIMT
Tsang M et al [85]	2017	The Netherlands	Specialized hospital clinic (Multicentric)	Prospective cohort	N/D	165	90.2	BMI	N/S	SLICC/ACR-DI

Table S2: Characteristics of individual evidence sources related to diabetes mellitus.

Source	Year of publication	Country	Setting	Type of study reported by the authors	Time frame	Number of participants	Percentage of women	Diabetes mellitus definition	Burden measures
Chanloung W et al[67]	2021	Thailand	Specialized hospital clinic	Cross-sectional	2 years	132	87	NICE Guidelines	APS Criteria Manifestations
Gergianaki I et al [69]	2021	Greece	Population-based registry	Cohort	44 month	399	91.2	N/S	BILAG
Gamal SM et al[71]	2021	Egypt	Specialized hospital clinic (multicenter)	Retrospective study	3 years	565	92.5	N/S	Lupus vasculitis
Sanchez-Perez H[64]	2020	Spain	Specialized hospital clinic (Multicentric)	Cross-sectional	N/S	195	95	N/S	CEC<br>cIMT
Ammar W et al[36]	2020	Egypt	Specialized hospital clinic	Cross-sectional	N/S	50	94	N/S	AoS<br>QAS<br>FMD<br>cIMT<br>PWV
Ajeganova S et al[65]	2020	Sweden	Specialized hospital clinic	Prospective cohort	7 years	77	90	History of diabetes mellitus prescription of antidiabetic drugs	cIMT
Quevedo-Abeledo JC et al[47]	2019	Spain	Specialized hospital clinic (Multicentric)	Cross-sectional	N/S	276	95	N/S	cIMT
Fernandez-Garces M et al[50]	2019	Spain	Specialized hospital clinic (Multicentric)	Cross-sectiona	10 years	335	100	Previous diagnosis or ≥2 measurements of fasting serum glucose ≥126mg/dL and/or use of antidiabetic medications	CVD
Mellor-Pita S et al[46]	2019	Spain	Specialized hospital clinic	Cross-sectional	N/S	47	100	Patient was treated with oral agents	Vitamin D levels

								or insulin or if fasting glucose was ≥ 126 mg/dl	
Petri MA et al[48]	2019	USA	Specialized hospital clinic	Cohort	1 year	1721	92	N/S	CVD
Baragetti A et al[21]	2018	Italy	Specialized hospital clinic	Prospective cohort	5 years	40	90	ADA criteria 2016	cIMT<br>SLEDAI-2K<br>SLICC/ACR-DI<br>PGA
Jordan-Gonzalez P et al[66]	2018	Puerto Rico/ USA	Hospitalized patients	Retrospective study	8 years	129	89.9	N/S	Risk of infections
Jiang MY et al[68]	2018	Taiwan	National population-based registry	Retrospective cohort	12 years	1317	91	At least 1 time of diagnosis of Diabetes Mellitus, or a prescription for anti-diabetic medication in the registry data.	ESRD incidence survival since ESRD diagnosis
Romero-Diaz J et al[3]	2018	Mexico	Specialized hospital clinic	N/S	7 years	95	0	N/S	Coronary artery calcium score
Wigren M et al[22]	2018	Sweden	Specialized hospital clinic	Cohort	9 years	470	88	N/S	CVD<br>cIMT
Vazquez-Del Mercado M[12]	2018	Mexico	Specialized hospital clinic	N/S	18 month	67	17.9	N/S	QRISK3
Garcia-Garcia P et al[35]	2017	Spain	Specialized hospital clinic	Cross-sectional	N/S	61	100	Patient was treated with oral agents or insulin or if fasting glucose was ≥ 126 mg/dl	Cystatin C
Falasinnu T et al[70]	2017	USA	Population-based registry	Cross-sectional	1 year	2036	86.2	ICD-10 as cause of death	Mortality

Table S3: Characteristics of individual evidence sources related to cardiovascular risk.

Source	Year of publication	Country	Setting	Type of study reported by the authors	Time frame	Number of participants	Percentage of women	Cardiovascular risk assessment	Percentage of patients with cardiovascular risk >10% or with atherosclerotic plaques	Associated factors
Zhu L et al[26]	2022	USA	Specialized hospital clinic (Multicentric)	Prospective cohort	15 years	366	97	QRISK3<br>FRS<br>mFRS<br>PREDICTS<br>ASCVD-ACC/AHA	N/S	N/S
Pesqueda-Cendejas K et al[39]	2022	Mexico	Specialized hospital clinic	Cross-sectional	3 years	176	100	Castelli index<br>Kannel index<br>TG/HDL-C ratio<br>Cardiometabolic index<br>LAP<br>hs-CRP	N/S	Mex-SLEDAI
Shaaban A et al[37]	2021	Egypt	Specialized hospital clinic	Cross sectional	N/S	30	76.7	cIMT	N/S	N/S
Patino-Trives AM et al[53]	2021	Spain	Specialized hospital clinic	Cross-sectional	2 years	80	90	cIMT<br>Plasma atherogenic index<br>ApoB/ApoA ratio	N/S	Positivity, titers and persistence of anti-dsDNA.<br>altered transcriptomic signatures in monocytes<br>NETosis-derived products
Weber BN et al[54]	2021	USA	Specialized hospital clinic	Prospective cohort	12 years	42	97	MFR<br>Coronary artery calcium score	N/S	Age<br>Disease duration
Sivakumaran J et al[14]	2021	Canada	Specialized hospital clinic	Retrospective cohort	47 years	1887	88	QRISK2<br>QRISK3<br>FRS<br>mFRS	QRISK2: 13<br>QRISK3: 20<br>FRS: 14.5<br>mFRS:17.5	N/S

								SLE Cardiovascular risk equation	SLE Cardiovascular risk equation: 48.5	
Sola-Rodriguez S et al[31]	2021	Spain	Specialized hospital clinic (multicentric)	Cross-sectional	5 month	77	100	Clustered cardiometabolic risk: Systolic blood pressure, fasting glucose, TG, HOMA-IR, TC/HDL-c, and hs-CRP	N/S	Relative Handgrip Strength<br>BMI
Campos-Lopez B et al[55]	2021	Mexico	Specialized hospital clinic	Cross-sectional	2 years	158	100	Glucose<br>Castelli index<br>Kannel index<br>tG/HDL-C ratio,<br>High TG/ low HDL-C<br>Cardiometabolic index<br>LAP score<br>hs-CRP<br>Waist to height ratio<br>Waist to hip ratio	N/S	Disease duration<br>Mex-SLEDAI
Chew C et al[18]	2021	Multi-site	Specialized hospital clinic (multicentric)	Cross-sectional	13 years	1163	88.9	HOMA-IR<br>Fasting insulin<br>Fasting glucose<br>HDL-C<br>TG<br>TC<br>Diastolic Blood Pressure<br>Systolic Blood pressure<br>Waist circumference	N/D	Vitamin D
Croca SC et al[56]	2021	UK	Specialized hospital clinic	N/S	N/S	100	95	Femoral and carotid IMT	36	TG<br>Prednisolone therapy<br>Persistent disease activity
Correa-Rodriguez M et al[57]	2021	Spain	Specialized hospital clinic (Multicentric)	Cross-sectional	N/S	264	89.4	Risk factors: Smoking, Hypertension, Dyslipidemia, Diabetes, Obesity<br>ABI	N/S	N/S

Quevedo-Abeledo JC et al[61]	2021	Spain	Specialized hospital clinic (Multicentric)	Cross-sectional	N/S	195	95	Atherogenic Index<br>CEC<br>Apo B:Apo A ratio	N/S	N/S
Pocovi-Gerardino G, et al[62]	2021	Spain	Specialized hospital clinic (Multicentric)	Cross-sectional	N/S	280	90.4	hs-CRP<br>homocysteine<br>Lipid profile<br>BMI<br>Fat mass percentage<br>ABI<br>Type II diabetes<br>Arterial hypertension	N/S	Med Diet adherence
Di Battista M et al[34]	2020	Italy	Specialized hospital clinic	Cohort	4 month	123	90.2	FRS<br>QRISK3<br>ASCVD-ACC/AHA	FRS: 23.6<br>QRISK3: 35.8<br>ASCVD-ACC/AHA: 13.8	N/S
Ammar W et al[36]	2020	Egypt	Specialized hospital clinic	Cross-sectional	N/S	50	94	AoS<br>QAS<br>FMD<br>cIMT<br>PWV	N/S	Disease duration<br>Arterial hypertension
Przywara-Chowaniec B et al[49]	2020	Poland	Specialized hospital clinic	N/S	45 month	38	89.4	cIMT<br>QRISK3	N/S	Disease duration
Masson W et al[51]	2020	Argentina	Specialized hospital clinic (Multicentric)	Cross-sectional	18 years	110	N/S	QRISK-3<br>mFRS	QRISK-3: 42.1<br>mFRS:54.8	N/S
Salvetti M[52]	2020	Italy	Specialized hospital clinic	N/S	10 month	33	100	FRS<br>PWV<br>cIMT	42	Weekly steroids dose
Drosos GC et al[13]	2020	Greece	Specialized hospital clinic	Prospective cohort	N/S	210	93.3	SCORE<br>FRS<br>ASCVD-ACA/AHA<br>PROCAM<br>mSCORE<br>mFRS	SCORE<br>: 7.6<br>mSCORE:9.0<br>FRS: 4.1<br>mFRS: 17.0<br>QRISK3:32.2	Age<br>Pack-years smoking<br>Cumulative corticosteroid dose

								QRISK3<br>cIMT	ASCVD: 13.2<br>PROCAM: 1.9%<br>Carotid* plaques were detected in 19.5%, femoral plaques in 14.3% and plaques at both sites in 10.6% of patients	
Bortoluzzi A et al[25]	2020	Italy	Specialized hospital clinic (Multicentric)	Cohort	2 years	20	100	cIMT	N/S	N/S
Casey KA et al[24]	2020	USA	Specialized hospital clinic (Multicentric)	Clinical trial	2 years	305	93.4	CEC<br>Lipid profile<br>GlycA<br>HOMA-IR<br>NET formation	N/S	N/S
Legge A[32]	2020	Canada	Specialized hospital clinic	Cross-sectional	12 month	100	92	ASCVD-ACC/AHA<br>TC/HDL ratio,<br>TG<br>Blood pressure<br>BMI	20	Moderate-vigorous physical activity (accelerometry)
Pocovi-Gerardino G[63]	2020	Spain	Specialized hospital clinic (multicentric)	Cross-sectional	22 month	139	90.6	Risk factors: Obesity, Diabetes mellitus, Arterial hypertension, Lipid profile<br>ABI	N/S	hs-CRP<br>Homocysteine
Lopez P et al[23]	2020	Spain	Specialized hospital clinic	Cohort	N/S	109	94	cIMT	33.9	CD16_ low-density granulocytes and monocyte-to-high density lipoprotein ratios
Sanchez-Perez H[64]	2020	Spain	Specialized hospital	Cross-sectional	N/S	195	95	CEC<br>cIMT	34	N/S

			clinic (Multicentric)					Apo B:Apo A ratio<br>Atherogenic Index		
Ajeganova S et al[65]	2020	Sweden	Specialized hospital clinic	Prospective cohort	7 years	77	90	cIMT	39	Lupus nephritis<br>Corticosteroids use<br>Arterial hypertension<br>TC/HDL ratio<br>LDL/HDL ratio
Rodriguez M et al[42]	2019	Spain	Specialized hospital clinic	Cross-sectional	N/D	78	92.3	cIMT	12.8	IDL and LDL net charge<br>Age
Mellor-Pita S et al[46]	2019	Spain	Specialized hospital clinic	Cross-sectional	N/S	47	100	PWV<br>cIMT<br>Risk factors prevalence: arterial hypertension, diabetes mellitus,hyperlipidemia, family history of cardiovascular, metabolic syndrome<br>hs-CRP<br>Homocysteine<br>Uric acid<br>Creatinine clearance<br>Microalbumin/creatinine	48	Vitamin D levels
Quevedo-Abeledo JC et al[47]	2019	Spain	Specialized hospital clinic (Multicentric)	Cross-sectional	N/S	276	95	SCORE<br>cIMT	36	Disease duration<br>SLICC/DI
Petri MA et al[48]	2019	USA	Specialized hospital clinic	Cohort	1 year	1721	92	ASCVD-ACC/AHA<br>FRS	N/S	N/S
Li Z et al[20]	2019	China	Specialized hospital clinic	Cross-sectional	3 years	98	100	Glucose<br>Creatinine<br>Uric acid<br>hs-CRP<br>TG<br>TC<br>HDL-C	N/S	Visceral adipose tissue

								LDL-C		
Fernandez-Garces M et al[50]	2019	Spain	Specialized hospital clinic (Multicentric)	Cross-sectiona	10 years	335	100	cIMT<br>ABI<br>BMD<br>Homocysteine<br>hs-CRP<br>Arterial hypertension<br>Smoking<br>Dyslipidemia<br>Sedentary lifestyle<br>Early menopause<br>Diabetes<br>Lipid profile	20.7	CVD
Chandrashekara S et al[59]	2019	India	Specialized hospital clinic	Cross-sectional	7 month	9	100	Atherogenic index of plasma	N/S	Uric acid levels
Rashad NM et al[60]	2019	Egypt	Specialized hospital clinic	Case–control	N/S	140	100	cIMT	N/S	Resistin<br>+299G/A gene polymorphisms
Jafri K et al[27]	2018	USA	Clinical and community-based sources (Multicentric)	Cross-sectional	N/S	157	93	FRS<br>mFRS<br>ASCVD-ACC/AHA	N/S	N/S
Yang XF et al [43]	2018	China	Specialized hospital clinic	Cross-sectional	10 month	90	100	ABI<br>PWV	N/S	Arterial hypertension<br>NLR<br>SLEDAI
Giannelou Met al [44]	2018	Greece	Specialized hospital clinic	Cross-sectional	N/S	150	94	cIMT	55.3	Hyperhomocysteinemia<br>MTHFR 677TT genotype
Wigren M et al[22]	2018	Sweden	Specialized hospital clinic	Cohort	9 years	470	88	cIMT	20.4	Fas<br>TNF receptor 1<br>TRAIL receptor 2<br>MMP-7
Garcia-Carrasco M[45]	2018	Mexico	Specialized hospital clinic	Cross-sectional	18 month	66	100	cIMT<br>mFRS	59	Levels of Treg cells

Vazquez-Del Mercado M[12]	2018	Mexico	Specialized hospital clinic	N/S	18 month	67	17.9	QRISK 3<br>cIMT<br>PWV<br>cfPWV<br>Arterial distensibility	QRISK3 : 15.2	Age<br>BMI
Baragetti A et al[21]	2018	Italy	Specialized hospital clinic	Prospective cohort	5 years	40	90	cIMT<br>FRS<br>SLE Cardiovascular Risk Equation	32	Levels of CD4þCCR5þ T cells<br>SLEDAI
Fox H et al[58]	2018	Germany	specialized hospital clinic	Prospective study	1 year	35	74.5	ABI<br>cIMT	N/S	N/S
Tziomalos K et al[33]	2017	Greece	Specialized hospital clinic	N/S	2 years	55	87.3	ABI<br>PWV<br>AIx	N/S	N/S
Garcia-Garcia P et al[35]	2017	Spain	Specialized hospital clinic	Cross-sectional	N/S	61	100	PWV<br>cIMT	N/S	Arterial hypertension<br>Metabolic syndrome<br>Hypertrigliceridemia<br>Smoking
Sanchez-Perez H et al[38]	2017	Spain	Specialized hospital clinic	Cross-sectional	5 years	87	95	cIMT	20%	N/S
Cypiene A et al[40]	2017	Lithuania	Specialized hospital clinic	Cross sectional	8 years	93	92.9	PWV<br>Alx<br>FMD	N/S	MBP
Lopez P et al[41]	2017	Spain	Specialized hospital clinic	Cohort	N/D	118	94.9	cIMT	32	anti-PON1 antibodies<br>anti-HDL antibodies
